# A C1q Domain Containing Protein from Scallop *Chlamys farreri* Serving as Pattern Recognition Receptor with Heat-Aggregated IgG Binding Activity

**DOI:** 10.1371/journal.pone.0043289

**Published:** 2012-08-15

**Authors:** Leilei Wang, Lingling Wang, Huan Zhang, Zhi Zhou, Vinu S. Siva, Linsheng Song

**Affiliations:** 1 Key Laboratory of Experimental Marine Biology, Institute of Oceanology, Chinese Academy of Sciences, Qingdao, China; 2 Graduate University, Chinese Academy of Sciences, Beijing, China; New England Biolabs, Inc., United States of America

## Abstract

**Background:**

The C1q domain containing (C1qDC) proteins refer to a family of all proteins that contain the globular C1q (gC1q) domain, and participate in a series of immune responses depending on their gC1q domains to bind a variety of self and non-self binding ligands.

**Methodology:**

In the present study, the mRNA expression patterns, localization, and activities of a C1qDC protein from scallop *Chlamys farreri* (CfC1qDC) were investigated to understand its possible functions in innate immunity. The relative expression levels of CfC1qDC mRNA in hemocytes were all significantly up-regulated after four typical PAMPs (LPS, PGN, β-glucan and polyI:C) stimulation. During the embryonic development of scallop, the mRNA transcripts of CfC1qDC were detected in all the stages, and the expression level was up-regulated from D-hinged larva and reached the highest at eye-spot larva. The endogenous CfC1qDC was dominantly located in the hepatopancreas, gill, kidney and gonad of adult scallop through immunofluorescence. The recombinant protein of CfC1qDC (rCfC1qDC) could not only bind various PAMPs, such as LPS, PGN, β-glucan as well as polyI:C, but also enhance the phagocytic activity of scallop hemocytes towards *Escherichia coli*. Meanwhile, rCfC1qDC could interact with human heat-aggregated IgG, and this interaction could be inhibited by LPS.

**Conclusions:**

All these results indicated that CfC1qDC in *C. farreri* not only served as a PRR involved in the PAMPs recognition, but also an opsonin participating in the clearance of invaders in innate immunity. Moreover, the ability of CfC1qDC to interact with immunoglobulins provided a clue to understand the evolution of classical pathway in complement system.

## Introduction

The C1q domain containing (C1qDC) proteins refer to a family of all proteins that contain the globular C1q (gC1q) domain, and participate in a series of immune responses depending on their gC1q domains to bind a variety of self and non-self binding ligands [1], [2], [3], [4]. gC1q is characterized by a jelly-roll topology consisting of a 10-stranded β-sandwich made up of two five-stranded anti-parallel β-sheets [5], [6]. Depending on the structural characteristics, C1qDC proteins are classified as C1q/C1q-like proteins containing the collagen or collagen-like region in the N-terminus, and globular head C1q proteins without the collagen region [1]. The members of this family are involved in several immune responses in innate immunity, such as pathogen recognition [7], activation of the complement system [8], mediating cell migration [9] and so on. Among all the C1qDC proteins, the mammalian complement C1q has been studied best.

As the target recognition unit in the complement system, complement C1q is formed by C1q A, B and C chains, and each chain contains a globular head (gC1q domain) in the N-terminal and a collagen region in the C-terminal [10]. Complement C1q binds IgM, IgG or C-reactive protein on surfaces through the globular heads, and then initiates the classical complement pathway [2], [11]. In addition, complement C1q is involved in other immunological processes, such as phagocytosis of bacteria, neutralization of retroviruses, cell adhesion, clearance of apoptotic cells and so on [2], [3]. The extreme versatility of C1q is due to the capacity of the gC1q domain to bind a variety of self and non-self ligands, including envelope proteins of retroviruses, β-amyloid fibrils, lipopolysaccharides (LPS), porins from Gram-negative bacteria, phospholipids and some acute phase reactants [2], [3], [12]. The gC1q domain has been considered as an extremely efficient and versatile charge pattern recognition domain, and the recombinant globular head A(ghA), B(ghB) and C(ghC) of C1q can function as the hexamer C1q to directly bind heat-aggregated IgG and IgM, envelope proteins of retroviruses and apoptotic cells [13]. Structural modeling of the globular heads in C1q revealed the predominance of the positive charged amino acid residues involved in the C1q-IgG interaction [12], and the predominant roles of ghB in the C1q-IgG interaction [6], [13], [14], [15]. The dominant role of Arg114 and the subsidiary roles of Arg129, Arg163 and His117 for human ghB in the C1q–IgG interaction were also confirmed by mutational study [12]. These cationic residues in ghB provide ionic interaction and form salt bridges with the residues of IgG, which play dominant roles in the C1q-IgG interaction [3].

Recently, many invertebrate C1qDC proteins have been identified from platyhelminths to cephalochordates, and some of them are found to be involved in immune response, including pathogen recognition [16], [17], microorganism agglutination [18], and mediating cell migration [9]. For example, the mRNA expression of AmphiC1q1 from *Cephalochordate amphioxus* was induced by LPS stimulation and Gram-negative bacteria challenge, and the recombinant AmphiC1q1 could bind LPS [17]. In molluscs, there were several reports about the up-regulation of mRNA expression of C1qDC proteins after the challenges of different pathogens [19], [20], [21], [22]. The recombinant AiC1qDC-1 from bay scallop *Argopecten irradians* could agglutinate fungi *Pichia pastoris* GS115 [18]. However, most of these invertebrate C1qDC proteins structurally lack collagen region in their C-terminal which is extremely important for the activation of complement system. Although current evidences support that some of these invertebrate C1qDC proteins are able to function as pattern recognition receptors (PRRs) to recognize a series of non-self ligands, the detailed mechanism of the immune recognition and the immune responses triggered by these invertebrate C1qDC proteins are still unknown.

In general, the activation of classical pathway is mainly mediated by the C1q-immunoglobulins interaction [23], [24], and cartilaginous fish is believed to be the evolutionarily earliest species having adaptive immunity, where C1q is involved in classical pathway. However, an orthologue of mammalian C1q was identified from lamprey *Lampetra japonicas* which acted as a lectin to bind Nacetylglucosamine (GlcNAc) and initiated the complement system through lectin pathway, indicating that C1qDC proteins could activate the complement system through binding pathogen associated molecular patterns (PAMPs) on the surface of pathogens in those animals lacking of immunoglobulins [7]. Recently, large numbers of C1qDC proteins have been identified from invertebrates, such as 50 C1qDC gene models in the *C. amphioxus* [17], [25], 7 in the purple sea urchin *Strongylocentrotus purpuratus* [26], and 168 in Mediterranean mussel *Mytilus galloprovincialis* [27]. In the evolutionary prospect, it is rather confusing that the functional capability of the invertebrate C1qDC proteins interaction with immunoglobulins.

A C1qDC protein (CfC1qDC) with LPS binding activity was identified previously from the Zhikong scallop *Chlamys farreri*, and the sequence was deposited in GenBank under accession No. EF536358 [16]. In the present study, the mRNA expression pattern, localization and protein activity were examined in order to (1) evaluate the functions of CfC1qDC in the innate immunity of *C. farreri* and (2) provide new insights into the evolution of the C1qDC proteins in complement system.

## Materials and Methods

### Immune Stimulation of Scallops

Adult scallops *C. farreri* with an average of 55 mm in shell length were collected from a farm in Qingdao, Shandong Province, China, and maintained in the aerated seawater at 15°C for a week before processing. Two hundred and forty scallops were employed for the PAMPs stimulation experiment. The scallops were randomly divided into 6 groups and each group contained 40 individuals. Five groups received an injection of 50 µL phosphate buffered saline (PBS, 0.14 M sodium chloride, 3 mM potassium chloride, 8 mM disodium hydrogenphosphate dodecahydrate, 1.5 mM potassium phosphate monobasic, pH 7.4), LPS from *Escherichia coli* 0111:B4 (Sigma-Aldrich, 0.5 mg ml^−1^ in PBS), peptidoglycan (PGN) from *Staphylococcus aureus* (Sigma-Aldrich, 0.8 mg ml^−1^ in PBS), β-glucan from *Saccharomyces cerevisiae* (Sigma-Aldrich, 1.0 mg ml^−1^ in PBS) and polyI:C (Sigma-Aldrich, 1.0 mg ml^−1^ in PBS), respectively. The untreated group was employed as blank group. After treatment, the scallops were returned to water tanks and 5 individuals were randomly sampled at 3, 6, 12, 24 and 48 h post-injection. The hemolymphs were collected, and centrifuged at 500×g, 4°C for 10 min to harvest the hemocytes for RNA preparation.

### Embryo Collection in Different Development Stages

All embryos and larvae were sampled from the Yixiang farm in Rongcheng, Shandong Province, China in April and May of 2011. Embryo or larvae from ten stages were identified microscopically, including Egg stage, zygote stage, 4-cell embryos stage, morula stage, blastula stage, gastrula stage, trochophore larvae, D-hinged larvae, veliger larvae and eye-spot larvae. Samples were collected and centrifuged at 300×g for 10 min, and preserved in Trizol (Invitrogen) for RNA extraction.

### RNA Isolation and cDNA Synthesis

Total RNA was isolated from the scallop samples using Trizol reagent (Invitrogen). The first-strand cDNA synthesis was carried out based on Promega M-MLV RT Usage information using the DNase I (Promega)-treated total RNA as template and oligo (dT)-adaptor as primer (Table 1). The reaction mixtures were incubated at 42°C for 1 h, and then terminated by heating at 95°C for 5 min. The cDNA mix was diluted to 1∶100 and stored at −80°C for following process.

**Table 1:** Names and sequences of primers used in this study.

Primer name	Sequence
Oligo (dT)-adaptor	5′-GGCCACGCGTCGACTAGTACT_17_-3′
**RT primers**	
P1 (forward)	5′-CGGTGGAGGTTACGATTTG-3′
P2 (reverse)	5′-TCCCGAGAAGGTCGCATA-3′
**β-Actin primers**	
P3 (forward)	5′-CAAACAGCAGCCTCCTCGTCAT-3′
P4 (reverse)	5′-CTGGGCACCTGAACCTTTCGTT-3′
**Elongation factor-1α primers**	
P5 (forward)	5′-ATCCTTCCTCCATCTCGTCCT-3′
P6 (reverse)	5′-GGCACAGTTCCAATACCTCCA-3′
**Recombination primers**	
P7 (forward)	5′-GCACCCACAGGTAACAATATCGT-3′
P8 (reverse)	5′-TTATCGTCCAGCCGCCGGAAAGT-3′
**Sequencing primers**	
T7 primer	5′-TAATACGACTCACTATAGGG-3′
T7t primer	5′-TGCTAGTTATTGCTCAGCGG-3′

### Real-time PCR Analysis of CfC1qDC mRNA Expression

CfC1qDC mRNA expression was examined by SYBR Green fluorescent quantitative real-time PCR (RT-PCR). Two gene-specific primers for CfC1qDC [16], sense primer P1 and reverse primer P2 (Table 1), were used to amplify a fragment of 289 bp, and the PCR product was sequenced to verify the specificity of RT-PCR. Two β-actin primers [16], sense primer P3 and reverse primer P4 (Table 1), were used to amplify a 94 bp fragment as an internal control to verify the successful transcription as well as to calibrate the cDNA template for corresponding scallop samples in PAMP stimulation experiment. Two elongation factor-1α primers [28], sense primer P5 and reverse primer P6 (Table 1), were used to amplify an 86 bp fragment as an internal control in embryonic development experiment. The SYBR Green RT-PCR assay was carried out in an ABI PRISM 7300 Sequence Detection System (Applied Biosystems) as described by Wang et al [29].

### Recombinant Expression of CfC1qDC and Purification of the Recombinant Protein

The cDNA fragment encoding the mature peptide of CfC1qDC was amplified using Taq polymerase (Promega) with specific primers P7 and P8 (Table 1). The PCR products were gel-purified and cloned into pEASY-E1 expression vector with a His tag in N-terminal (Transgen, China). The recombinant plasmid (pEASY-E1-CfC1qDC) was transformed into Trans-T1 phage resistant chemically competent cell (Transgen). The forward positive clones were screened by PCR using vector primer T7 and recombination primer P8 (Table 1), and further confirmed by nucleotide sequencing with vector primer T7 and T7t (Table 1). The valid recombinant plasmid (pEASY-E1- CfC1qDC) was extracted and transformed into *E. coli* BL21 (DE3)-Transetta (Transgen). The pET-32a vector without insert fragment was selected as negative control, which could express a thioredoxin with a His tag in the prokaryotic expression system. Positive transformants were incubated in LB medium (containing 50 mg ml^−1^ ampicillin) at 37°C with shaking at 220 rpm. When the culture mediums reached OD_600_ of 0.5–0.7, the cells were incubated for 4 additional hours with the induction of IPTG at the final concentration of 1 mmol L^−1^. The recombinant protein CfC1qDC (designated rCfC1qDC) were purified, refolded, verified and quantified as described by Zhang [30]. The obtained protein was stored at −80°C for subsequent experiments.

### Preparation of Antibody and Western Blotting Analysis

To prepare antibody, the renatured protein of rCfC1qDC was dialyzed continuously for 12 h against ddH_2_O and then concentrated through freeze-drying. Six-week old rats were immunised by rCfC1qDC to acquire polyclonal antibody according to the previous description [31].

After SDS-PAGE, the samples of whole cell lysate (4 h after IPTG added) were electrophoretically transferred on to a 0.45 mm pore nitrocellulose membrane at 200 mA for 5 h. The membrane was blocked with PBS containing 3% BSA at 37°C for 1 h, and incubated with antibody (diluted 1∶1000 in PBS) at 37°C for 1 h. After washed three times with PBS containing 0.05% Tween-20 (PBS-T), the membrane was then incubated with goat-anti-rat Ig-alkaline phosphatase conjugate (Southern Biotech) diluted 1∶4000 in PBS at 37°C for 1 h, and then washed three times again with PBS-T. Protein bands were stained with freshly prepared substrate solution (100 mM NaCl, 100 mM Tris and 5 mM MgCl_2_, pH 9.5) containing nitroblue tetrazolium (NBT, Sigma) and 5-bromo-4-chloro-3-indolyphosphate (BCIP, Sigma) for 5 min and stopped by washing with distilled water. Rat pre-immune serum was used as negative control.

### Immunofluorescence Detection of CfC1qDC in Tissues

The slides of hemocytes were prepared according to the previous method [32]. Seven tissues of *C. farreri* including gill, adductor muscle, gonad, hepatopancreas, mantle and kidney, were fixed in Bouin’s fluid for 24 h. The fixed tissues were rinsed, dehydrated and embedded in paraffin wax. Longitudinal sections (5 µm) were cut using a Leica RM2016 microtome and mounted on slides, and then subjected to deparaffination in xylene and rehydration in diluted ethanol series. The tissue sections were washed in PBS-T, and then antigen retrieval was performed by heating the tissue sections in water bath at 120°C for 10 min.

The slides were blocked by incubating in PBS containing 3% BSA for 1 h. Then 20 µl antibody of rCfC1qDC (diluted 1∶ 1000 in PBS) was added and incubated at 37°C for 1 h in a moisture chamber. After three times’ washing with PBS-T, the slides were incubated at 37°C for 1 h with Alexa Fluor® 488 dye conjugated goat-anti-rat immunoglobulins serum (Invitrogen) diluted at 1∶ 500 with PBS containing 1 µg ml^−1^ Evan’s blue dye (EBD, Fluka) as the counterstain. Finally, the slides were washed three times and mounted in buffered glycerin for observation by fluorescence microscope (Olympus). Rat pre-immune serum was used as negative control.

### PAMPs Binding Assay

The PAMPs binding assay was performed according to previous report with modification [33]. Briefly, 20 µg of LPS from *E. coli* (Sigma-Aldrich), PGN from *S. aureus* (Sigma-Aldrich), β-glucan (Glu) from *S. cerevisiae* (Sigma-Aldrich) and polyI:C (Sigma-Aldrich) in 100 µL PBS were used to coat 96-well microtiter plate (Costar 92592, USA). The wells were then blocked with 3% BSA in PBS at 37°C for 1 h. After washing with PBS-T, 1/2-fold serial dilution concentrations of rCfC1qDC in TBS buffer (50 mM Tris-HCl, 50 mM NaCl, pH 7.6) were added in the presence of 5 mM CaCl_2_ and 0.1 mg ml^−1^ BSA, and the highest protein concentration was adjusted to 150 µg/ml. The same concentration of recombinant thioredoxin (rTrx) was used as control, and the wells with 100 µL of TBS were used as blank. After incubating at 18°C for 3 h and washing for three times, 100 µL of rat immune serum of rCfC1qDC (diluted to 1∶1000) was added and incubated at 37°C for 1 h. The plate was washed again and 100 µL of goat-anti-rat Ig-alkaline phosphatase (AP) conjugate (Southern Biotech, diluted to 1∶4000) was added and incubated at 37°C for another 1 h. After the last rinse, 100 µL of 0.1% (w/v) p-nitrophenyl phosphate (pNPP, Sigma) in 50 mM carbonate bicarbonate buffer (pH 9.8) containing 0.5 mM MgCl_2_ was added and incubated at room temperature in dark for 30 min. The reaction was stopped by 2 M NaOH and the absorbance was measured at 405 nm. Non-immune rat serum instead of immune serum as first antibody was set as negative control. Each experiment was repeated in triplicate. Samples with P/N = P (sample) −B (blank)/N (negative) −B (blank) >2.1 were considered positive.

### Heat-aggregated IgG Interacting Assay

Heat-aggregated IgG was prepared according to methods described previously with modification [34]. In brief, purified human IgG (Sigma–Aldrich) was prepared to a concentration of 500 µg/ml with TBS-NTC buffer (50 mM Tris-HCl, pH 7.6, 150 mM NaCl, 0.05% W/V NaN_3_, 0.05% V/V tween-20 and 5 mM CaCl_2_). After heating at 63°C for 30 min, the IgG was cooled on ice immediately and stored at 4°C for subsequent experiment.

The interaction of rCfC1qDC with heat-aggregated IgG was analyzed according to previous report with modification [12]. The rCfC1qDC was coated in microtiter wells within 0.2 M carbonate buffer (pH 9.6) at different concentrations (0.078, 0.156, 0.313, 0.625, 1.25, 2.5, and 5 µg/well) overnight at 4°C. The same concentration of rTrx was used as control. The wells were washed and then blocked with PBS containing 3% (w/v) BSA for 2 h. After three rounds of washing, the wells were incubated with heat-aggregated human IgG (10 µg/well) in PBS-T at 18°C for 2 h. The bound IgG was detected using goat-anti-human IgG-AP conjugate (Bioss, China) and pNPP (Sigma–Aldrich) as described previously. Each experiment was repeated in triplicate.

### Inhibition Assay of LPS in the Interaction between CfC1qDC and Heat-aggregated IgG

The rCfC1qDC (suspended in 0.2 M carbonate buffer, pH 9.6) was used to coat the microtiter wells at different concentrations (0.078, 0.156, 0.313, 0.625, 1.25, 2.5 and 5 µg/well) by incubation at 4°C overnight. The microtiter plates were washed and incubated with PBS containing 3% (w/v) BSA for 2 h. After three rounds of washing, one group of the wells was blocked with LPS (20 µg/well) in PBS, and the other group was added only the PBS, and incubated for 3 h at 18°C. Subsequently, the wells were washed three times and incubated with heat-aggregated human IgG (10 µg/well) in PBS-T for 2 h at 18°C. After the washing, bound IgG was detected using goat-anti-human IgG-AP conjugate (Bioss, China) and pNPP (Sigma–Aldrich) as described previously. The wells coated with rTrx were used as control, and each experiment was repeated in triplicate.

### Phagocytosis Assay

Phagocytosis assay was performed according to previous method with modification [35]. Briefly, Hemolymph was collected from five scallops with a syringe, and mixed immediately with equal volume of pre-chilled anticoagulant (Tris-HCl 50 mM, glucose 2%, NaCl 2%, EDTA 20 mM, pH 7.4), and then centrifuged at 800 g for 10 min. The hemocytes were resuspended at 1×10^6^ cells/mL^−1^ in TBS buffer, TBS buffer with rCfC1qDC (100 µg mL^−1^), and TBS buffer with rTrx (100 µg mL^−1^), respectively. Then FITC-labeled *E. coli* was added into hemocyte suspension at the final concentration of 1×10^8^ cells/mL^−1^. After incubation at 18°C for 45 min, 50 µL of hemocyte suspension was smeared onto glass slide treated with poly-L-lysine, and followed by incubation for 30 min for attachments of hemocytes to form a monolayer. Subsequently the fluorescence of nonphagocytosed bacteria was quenched by the trypan blue solution (2 mg/mL^−1^ in PBS) (Amresco). After washing with PBS for five times, the hemocytic phagocytosis was examine under fluorescent microscope (Olympus). Two hundred hemocytes on each slide were counted. Phagocytic rate (PR) and phagocytic index (PI) representing the phagocytic activities were expressed as following: PR = (phagocytic hemocytes)/(total hemocytes)×100%; PI =  average number of bacteria in phagocytic hemocyte. For each treatment, assay was performed in four different slides for statistic analysis.

### Sequence Analysis

The amino acid sequence of CfC1qDC was analyzed using the BLAST algorithm (http://www.ncbi.nlm.nih.gov/blast) and the Expert Protein Analysis System (http://www.expasy.org/). The protein domains were revealed by the simple modular architecture research tool (SMART) version 4.0 (http://www.smart.emblheidelberg.de/). The presumed tertiary structures of gC1q domain of CfC1qDC and human complement C1q-B (NP_000482) were established using the SWISS-MODEL prediction algorithm (http://swissmodel.expasy.org/) [36], [37] and displayed by PyMOL Viewer 1.3 (http://www.pymol.org). The ClustalW Multiple Alignment program (http://www.ebi.ac.uk/clustalw/) was used to create the multiple sequence alignment. An unrooted phylogenetic tree was constructed based on the sequence alignment by the neighbor-joining (NJ) algorithm using the Mega 3.1 program [38]. The reliability of the branching was tested by bootstrap resampling (1000 pseudo-replicates).

### Statistical Analysis

All date was given as means ± S.E. The data was subjected to one-way analysis of variance (one-way ANOVA) followed by a multiple comparison (S-N-K). Differences were considered significant at *P*<0.05.

## Results

### The mRNA Expression Profiles in the Hemocytes of Scallops After PAMP Stimulations

**Figure 1:**
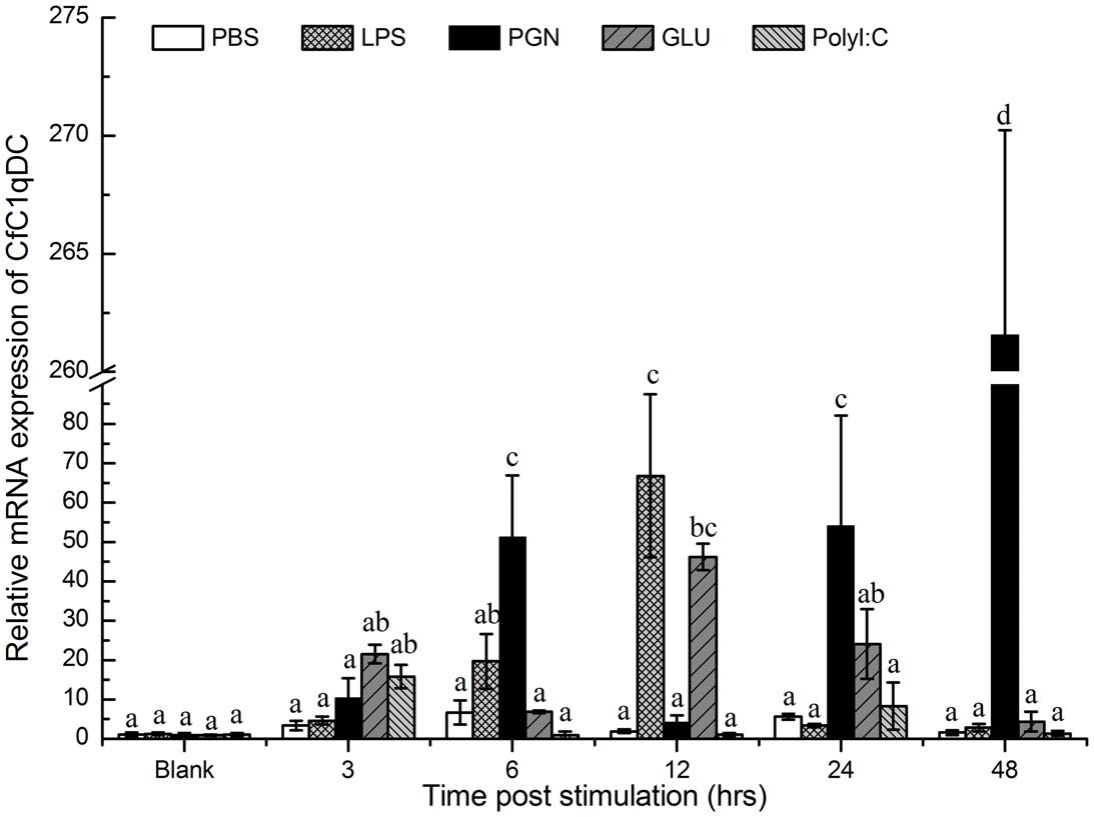
Temporal expression of the CfC1qDC transcripts in hemocytes after PAMPs challenge. Vertical bars represent the mean ± S.E. (N = 5).

Real-time PCR was used to monitor the mRNA expression of CfC1qDC transcripts in hemocytes of adult animals stimulated by four typical PAMPs (Figure 1). In the LPS stimulated group, the mRNA expression of CfC1qDC was significantly up-regulated at 12 h (66.7-fold, *P*<0.05) after stimulation, and then decreased to the original level gradually. In the PGN stimulated group, after a significant increase at 6 h (51.2-fold, *P*<0.05) post-stimulation, the mRNA expression of CfC1qDC decreased at 12 h, and then gradually increased to the highest expression level at 48 h (261.6-fold, *P*<0.05). In the β-glucan stimulated group, the expression of CfC1qDC reached to the highest level at 12 h (46.2-fold, *P*<0.05) post stimulation, and then dropped back to the original level gradually. In contrast, the mRNA expression of CfC1qDC in polyI:C stimulated group didn’t change greatly. In the control group, there was no significant change of CfC1qDC expression during the whole experiment after PBS injection.

### The mRNA Expression Pattern of CfC1qDC during Embryonic Development

**Figure 2:**
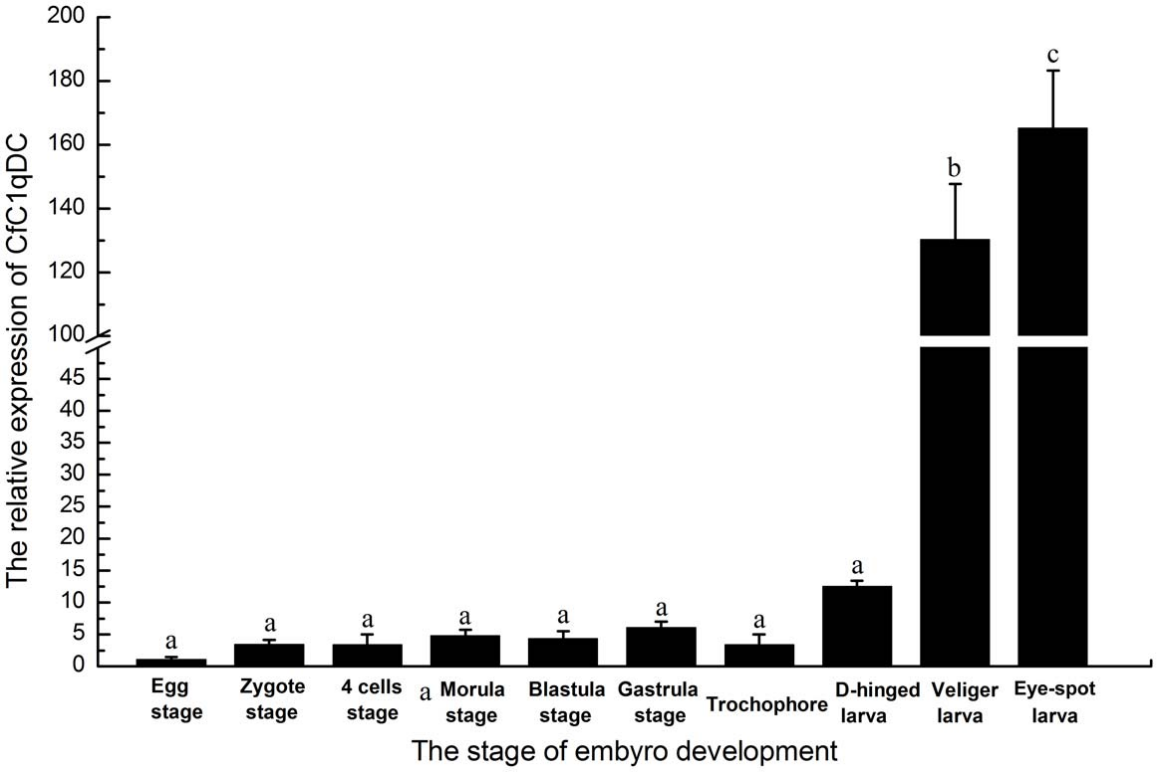
Temporal expression of the CfC1qDC transcripts in the different stages of scallop embryo development. Vertical bars represent the mean ± S.E. (N = 5).

In order to elucidate the expression pattern of CfC1qDC in the embryonic development, its mRNA expression level was monitored by Real-time PCR. The mRNA transcript of CfC1qDC was detected in all the stages of development (Figure 2). The expression level of CfC1qDC was up-regulated slightly in the early stage of embryonic development. From the stage of D-hinged larva, the expression level of CfC1qDC began to increase rapidly, and reached to the highest level in the stage of eye-spot larva, which was 165.2-fold compared with that in egg stage (Figure 2).

### The Recombinant Protein of CfC1qDC and its Antibody

**Figure 3:**
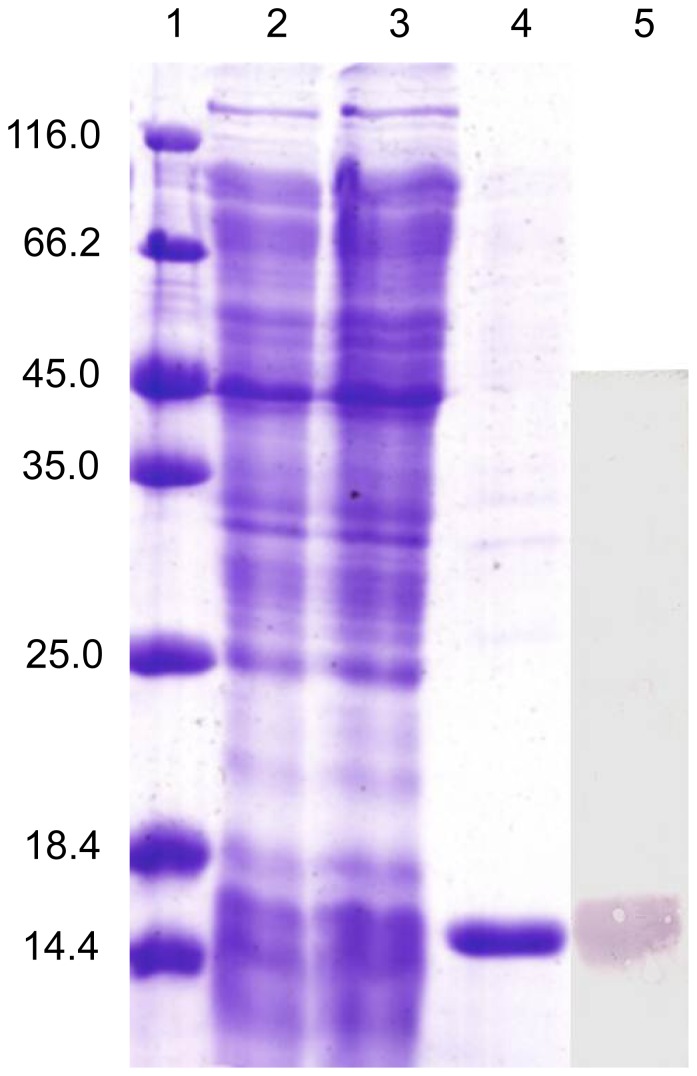
SDS-PAGE and western-blot analysis of rCfC1qDC. Lane 1: protein molecular standard (kDa); lane 2: negative control for rCfC1qDC (without induction); lane 3: induced rCfC1qDC; lane 4: purified rCfC1qDC; lane 5: western blot based on the sample of line 3.

The recombinant plasmid (pEASY-E1-CfC1qDC) was transformed into *E. coli* BL21 (DE3)-Transetta (Transgen). After IPTG induction for 4 h, the whole cell lysate was analyzed by SDS-PAGE, and a distinct band was revealed with a molecular weight of 17 kDa (Figure 3), which was in accordance with the previous predicted molecular weight 17.2 kDa [16]. The concentration of the purified rCfC1qDC protein was adjusted to 860 µg mL^−1^ by BCA method.

The purified protein was used to immunise rats, and the immune serum was collected. Western blotting was carried out to examine the specificity of antibody, and a clear reaction band with high specificity was revealed (Figure 3). As negative control, no visible reaction band was detected in group of rat pre-immune serum (data not shown).

### Immunofluorescence Detection of CfC1qDC in Tissues

**Figure 4:**
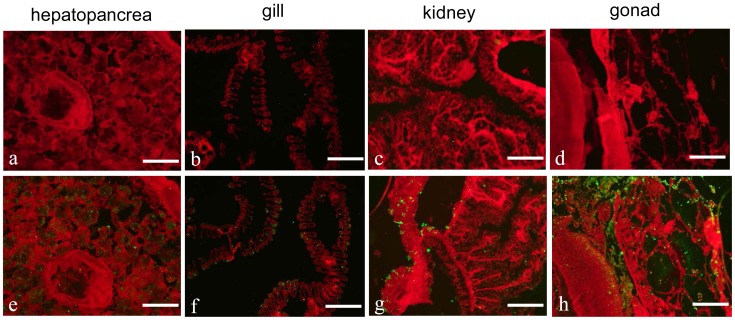
Localization of endogenous CfC1qDC in different tissues using rat polyclonal antiserum. Binding of antibody was visualized by Alexa Fluor® 488 dye conjugated secondary antibody (green), and the whole tissues were stained with EBD (red). Hepatopancreas (a and e); gill (b and f); kidney (c and g); gonad (d and h). All the bars represent 100 µm.

Localization of endogenous CfC1qDC in different tissues was observed by the method of immunofluorescence. CfC1qDC was detected in the hepatopancreas (Figure 4e), gill (Figure 4f), kidney (Figure 4g) and gonad (Figure 4h) of the scallops, while no fluorescence signal was observed in other tissues, including mantle, hemocytes and muscle (data not shown). No fluorescence signal was observed in all the negative control groups (Figure 4a, b, c and d).

### PAMPs Binding Activity of rCfC1qDC

**Figure 5:**
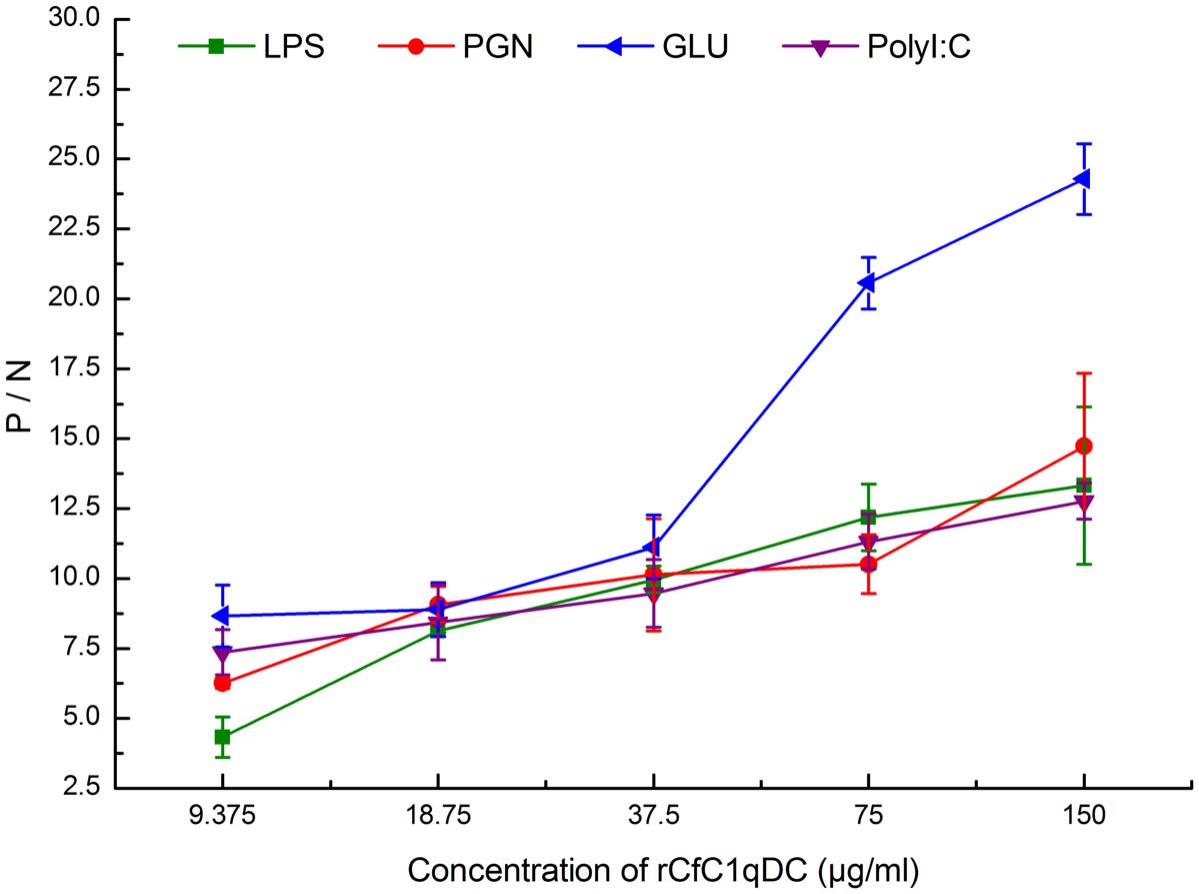
ELISA analysis of the interaction between rCfC1qDC and the PAMPs. Plates were coated with various PAMPs, and then incubated with several concentrations of rCfC1qDC and rTrx at 18°C for 3 h. After incubated with rat polyclonal antiserum, the interaction was detected with goat–anti-rat Ig-alkaline phosphatase conjugate at 405 nm. Samples with P/N >2.1 were considered positive. Results are representative of average three such experiments.

The activity of rCfC1qDC to bind different PAMPs was recorded as P/N value at 405 nm, and the samples with P/N >2.1 were considered as positive. The recombinant CfC1qDC protein exhibited affinity to LPS, PGN, β-glucan and polyI:C, and the binding ability was dose-dependent (Figure 5). As control, the P/N of rTrx groups were all around 0.9, indicating that rTrx could not bind any tested PAMPs (data not shown).

### The Interaction between rCfC1qDC and Human Heat-aggregated IgG

**Figure 6:**
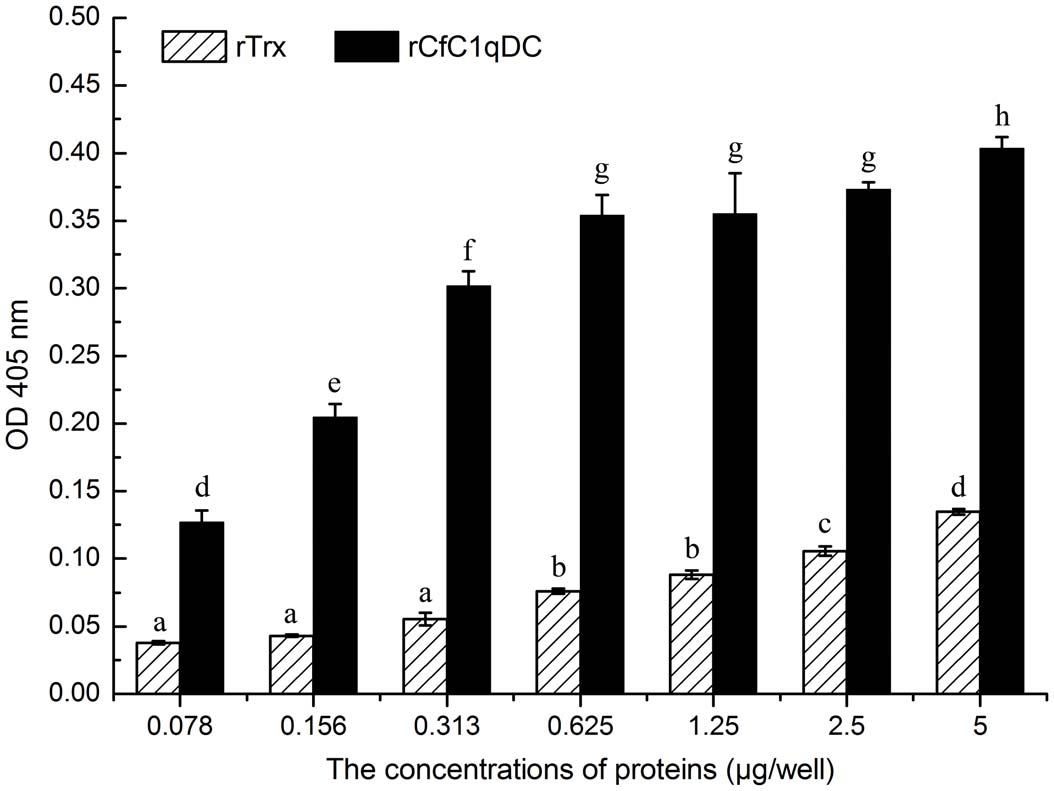
ELISA analysis of the interaction between rCfC1qDC and human heat-aggregated IgG. Plates were coated with various PAMPs several concentrations of rCfC1qDC and rTrx, and then incubated with human heat-aggregated IgG. The interaction was detected with goat–anti-human Ig-alkaline phosphatase conjugate at 405 nm. Results are representative of average three such experiments.

**Figure 7:**
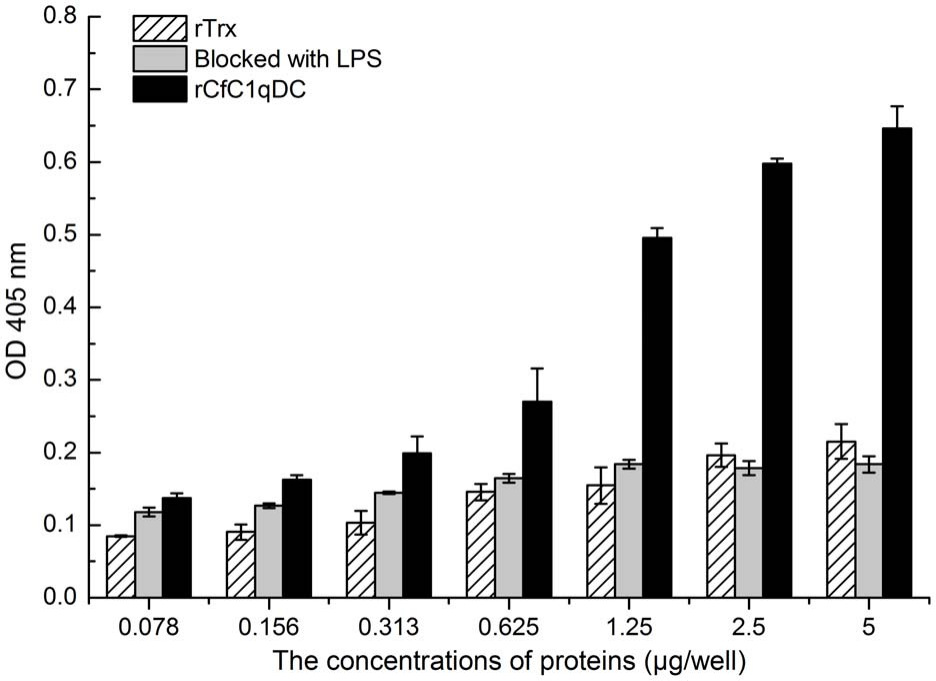
The inhibition effect of LPS in the rCfC1qDC and human heat-aggregated IgG interaction. Results are representative of average three such experiments.

The absorbance of rCfC1qDC group rose rapidly with the increase of protein concentration, indicating that rCfC1qDC could bind human heat-aggregated IgG in a dose-dependent manner (Figure 6). However, the absorbance was decreased obviously when wells were blocked with LPS (Figure 7). The values of the control protein were very low in the same concentration.

### Phagocytic Activity of Scallop Hemocytes Enhanced by rCfC1qDC

**Figure 8:**
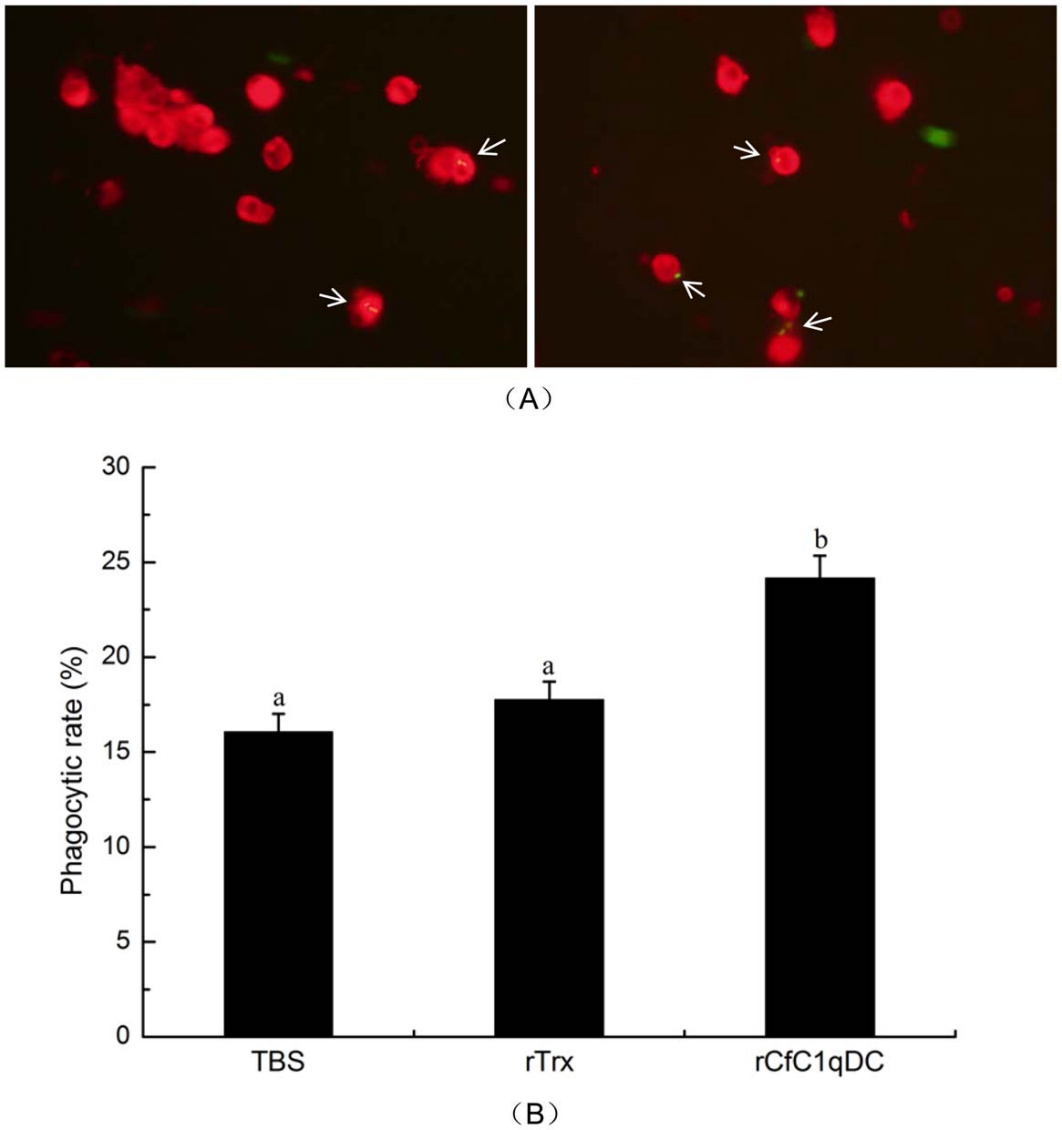
Phagocytosis enhanced by rCfC1qDC. The hemocytes were resuspended with TBS buffer, rTrx in TBS buffer and rCfLec-1 in TBS buffer, respectively. Then E. coli was added into each hemocytes suspension for 45 min. The mixture was mounted onto a glass slide and incubation for 30 min to allow attachments of hemocytes to form a monolayer. After the fluorescence of nonphagocytosed bacteria was quenched by the trypan blue, hemocytes on each slide were counted (A). Phagocytic rate (PR) = (phagocytic hemocytes)/(total hemocytes)×100% (B). For each treatment, assay was performed in four different slides for statistic analysis. Bar  = 10 µm.

rCfC1qDC could significantly enhance the phagocytic activity of scallop hemocytes (Figure 8). Under microscope, the phagocytized *E. coli* was clearly observed in the cytoplasm of the hemocytes (Figure 8A). The phagocytic rate of hemocytes treated with rCfC1qDC increased significantly (*P*<0.05) compared with the treatments of TBS and rTrx. The phagocytic rate of rCfC1qDC group was 24.1%, while those in TBS and rTrx groups were 16.0% and 17.8%, respectively (Figure 8B). The phagocytic index was around 1.6, and it did not change significantly in all the three groups (date not shown).

### Tertiary Structures and Phylogenetic Analysis

**Figure 9:**
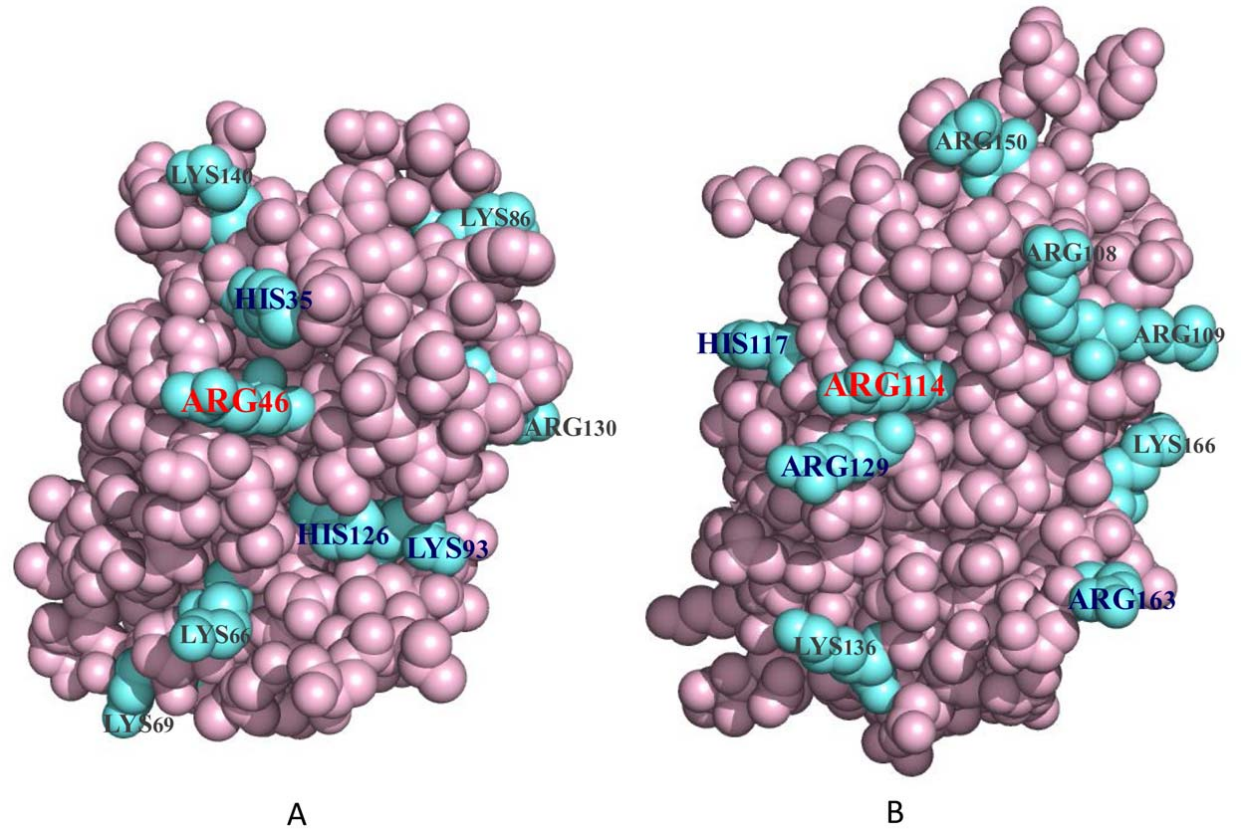
The predicted spatial structures of gC1q domains. The gC1q domains from CfC1qDC (A) and globular head B of human complement C1q (B) were predicted by SWISS-MODEL program. The blue molecules represent the cationic amino acid residues on the surface of gC1q domains.

The potential tertiary structures of gC1q domains in CfC1qDC and ghB of human complement C1q were established using the SWISS-MODEL prediction algorithm based on the template 2jg8B and 1pk6B, respectively. As shown in Figure 9, the spatial location of Arg46 in the gC1q domain of CfC1qDC protein was similar to that of Arg114 in ghB, and the cationic amino acid residues His35, Lys93 and His126 in CfC1qDC were located at almost the same position as that of the cationic residues in ghB.

**Figure 10:**
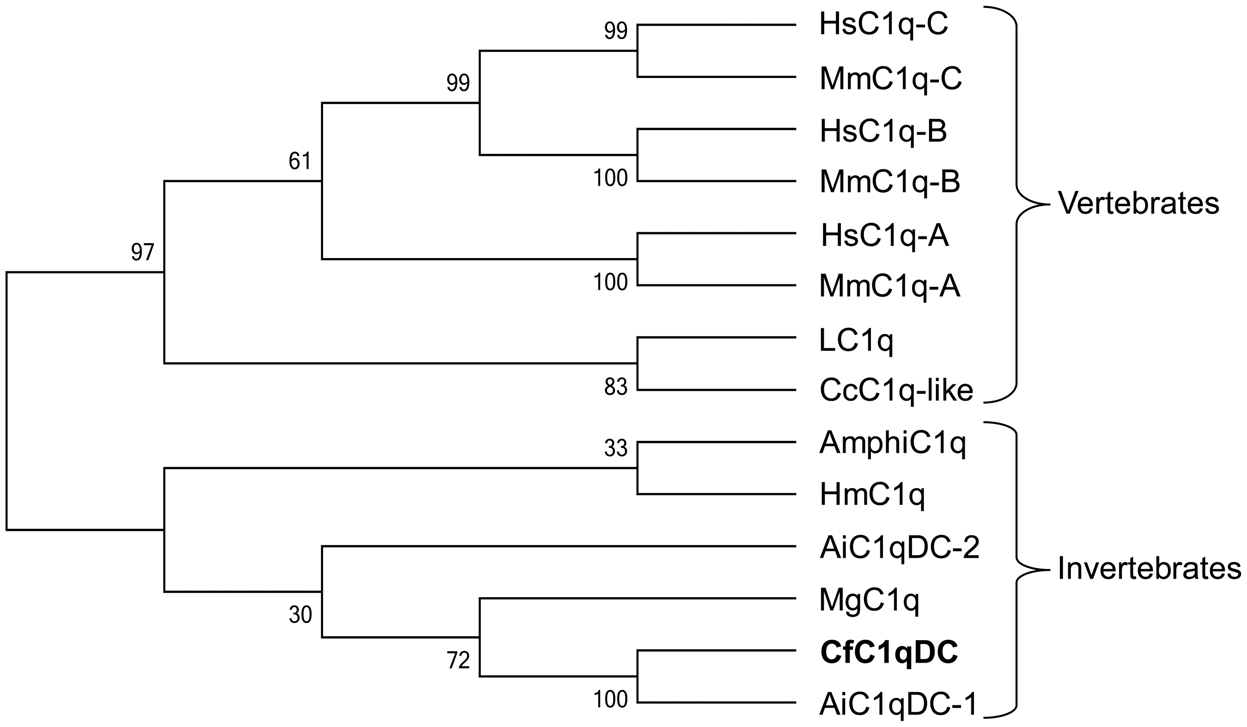
Phylogenetic tree of gC1q domains from different C1qDC proteins. The tree is constructed by the neighbor-joining (NJ) algorithm using the Mega 3.1 program based on the multiple sequence alignment by ClustalW. The reliability of the branching is tested by bootstrap re-sampling (1000 pseudo-replicates). The gC1q domain sequences used for phylogenetic analysis are as follows: Human C1q-A (**NP_057075**), Human C1q-B (**NP**_**000482**), Human C1q-C (**NP_758957**), Mouse C1q-A (**NP_031598**), Mouse C1q-B (**NP_033907**), Mouse C1q-C (**NP_031600**), LC1q (**BAD22833**), CcC1q-like (**BAD22535**), AmphiC1q (**ACH95418**), HmC1q (**ACC69183**), AiC1qDC-2 (**GU475114**), MgC1q (**FN563147**), CfC1qDC (**EF536358**) and AiC1qDC-1 (**GU475113**).

A phylogenetic tree was constructed using neighbor-joining method with 1000 bootstrap test based on the multiple alignments of gC1q domains from CfC1qDC and other C1qDC proteins (Figure 10). Two distinct groups, representing vertebrate and invertebrate C1qDC proteins, were separated in the tree, and CfC1qDC were clustered into invertebrate subgroup.

## Discussion

In vertebrates, complement C1q recognizes a variety of self and non-self ligands through the gC1q domain [2], [3], [12], and is consequently involved in a series of immunological processes, such as activation of complement system, phagocytosis of invaders and neutralization of retroviruses [2], [3], [11]. To our knowledge, no complement C1q has been reported from invertebrate, while lots of C1qDC proteins have been identified from platyhelminths to cephalochordates. As the information about the function of invertebrate C1qDC proteins is limited, the evolutionary relationship between invertebrate C1qDC proteins and vertebrate complement C1q remains unclear yet. In the present study, the mRNA expression pattern, localization, and protein activity of CfC1qDC were investigated in order to reveal its function in innate immunity, and provide new insights into the evolution of C1qDC proteins in complement system.

Immune recognition is the first and crucial step in the innate immunity, which discriminates self from the potentially harmful non-self and activates a series of immune response [39]. C1qDC proteins acquire the capacity to engage a variety of non-self ligands via the gC1q domain [2], [3], [12]. In invertebrates, several members of this family have been characterized, and their mRNA expression could be induced by the PAMPs stimulation or microbes challenge [16], [17], [18], [40]. Similarly, the mRNA expression of CfC1qDC in the present study was significantly up-regulated after scallops were stimulated by LPS, PGN, β-glucan or polyI:C. As bivalves possess the open circulatory system, and the circulating hemocytes play crucial roles both in cellular and humoral immunity [40], [41], the significant up-regulation of transcripts indicated that CfC1qDC was involved in the immune response induced by these PAMPs. In vertebrates, complement C1q binds directly to the surfaces of many Gram-negative bacteria through the gC1q domain without the help of antibody, and this binding is mediated by lipid A, LPS and porins [3], [42]. In the present study, CfC1qDC was detected to bind LPS, PGN, β-glucan and even the polyI:C, indicating that CfC1qDC could serve as a PRR to recognize and bind various PAMPs. Although mRNA expression of CfC1qDC was not up-regulated significantly after polyI:C injection, CfC1qDC could also bind polyI:C directly, which suggested that CfC1qDC might be involved in the recognition of RNA virus in scallop immune system.

Opsonins are host-derived proteins that can increase the efficiency of phagocytosis and diversify the functional repertoire of phagocytes [41], [43]. In mammals, complement C1q functions as an opsonin, which can coat bacteria and enhance the uptake of bacteria by phagocytic cells [44], [45]. In invertebrates, although lots of C1qDC proteins have been identified, their functions in bacteria clearance are still far from well understood. In the present study, rCfC1qDC could bind PAMPs from microbes and enhance the ability of hemocyte to phagocytize bacteria *in vitro*. The PRR mediated phagocytosis has also been reported in scallop, such as C type lectin (CfLec-1) in Zhikong scallop *C. farreri* which could mediate the opsonization against *E. coli* [39]. These results indicated that CfC1qDC could not only function as a PRR to participate in immune recognition, but also trigger opsonization like complement C1q. It is known that the collagen region of complement C1q is responsible for the mediation of enhanced phagocytosis [46]. Because there was no collagen region in the C-terminal of CfC1qDC, it was suggested that the opsonization mechanism of CfC1qDC might be different from complement C1q.

Ontogenesis of immune system is of prime importance in scallop embryo development. During the bivalve embryo development, the major maturation events leading to immunocompetence occur between D-hinged larvae and veliger larvae, and hemocyte generation/proliferation as well as induction of immune related genes are concomitant in these stages [47]. Because CfC1qDC functioned as either PRRs in immune recognition or an opsonin in pathogen clearance, the progressive increase of CfC1qDC transcription from D-hinged larvae to eye-spot larvae manifested that CfC1qDC should be involved in the immune defense during ontogenesis. In healthy adult scallop, CfC1qDC protein could be specifically detected in hepatopancreas, gonad, gill and kidney by immunofluorescence technique. Because hepatopancreas and gonad are considered to be the important organs synthesizing proteins executing in immune system [48], and play central roles in bivalve defense [49], the synthesis of CfC1qDC in these organs suggested its involvement of immunity. Gill is the organ responsible for seizing oxygen and alga from the seawater, and the presence of CfC1qDC protein in this organ may be because of its frequent contact with pathogens. Existence of CfC1qDC protein in kidney was in accord with its higher mRNA expression level in kidney from our previous report [16]. As an excretory organ, scallop kidney connects with pericardial cavity, and it is easy to be invaded by pathogens. The CfC1qDC protein in kidney may perform crucial roles in preventing pathogen invasion.

As the target recognition unit in complement system, the interaction of complement C1q with immunoglobulins via the gC1q domain is the basis of the activation of classical pathway in vertebrates [2], [11]. It is still not clear when the C1qDC proteins obtain the ability to interact with immunoglobulins in evolution, and no invertebrate C1qDC protein has been reported to interact with immunoglobulins up to now. In the present study, rCfC1qDC bound heat-aggregated IgG in a dose-dependent manner which was similar as the globular head of vertebrate C1q [13], strongly indicating that the scallop CfC1qDC protein had the ability to bind immunoglobulins. It is well known that the classical pathway is only present in the species with immunoglobulins, and it is activated predominantly by immunoglobulins via C1q binding [50]. Because CfC1qDC takes the ancient position in the phylogenetic tree, its ability to bind heat-aggregated IgG implied that the ancient invertebrate C1qDC proteins had obtained the capability to bind immunoglobulins. However, it’s participation in classical pathway might not be established until in cartilaginous fish. At the same time, it was reported that C1qDC protein from lamprey *L. japonicas*, bound PAMPs as a lectin instead of immunoglobulins and initiated the complement system through lectin pathway [7]. Considering this fact, CfC1qDC and other invertebrate C1qDC proteins [16], [17], [18] could serve as PRRs to recognize PAMPs, it is believed that C1qDC proteins can activate ancient complement system by lectin pathway before the evolution of immunoglobulins.

The interaction of the complement C1q with IgG is predominantly supported by the positive charged amino acid residues on the gC1q domain, which provide an ionic interaction and form salt bridges with the residues of IgG [3], [12]. In ghB of human complement C1q, the Arg114 is confirmed to play dominant role, and Arg129, Arg163 and His117 play subsidiary roles in the C1q–IgG interaction [12]. The tertiary structures of gC1q domain of CfC1qDC protein and ghB were compared by using the SWISS-MODEL prediction algorithm, and the spatial location of Arg46 on the gC1q domain of CfC1qDC protein was found to be similar as that of Arg114 on ghB, implying that Arg46 of CfC1qDC protein might play the same dominant role in the interaction with IgG. There were also three cationic amino acid residues (His35, Lys93 and His126) located at the similar positions as the subsidiary cationic residues in ghB. The similarity of the spatial distribution of these cationic residues in these two gC1q domains indicated that CfC1qDC protein might bind the IgG through these surface cationic amino acid residues. Complement C1q also recognizes LPS mainly through the cationic amino acid residues on its gC1q domain to bind the anionic phosphate groups of LPS [4]. In the present study, LPS could inhibit the CfC1qDC-IgG interaction, suggesting that both LPS and IgG might be share the same binding region and cationic amino acid residues for binding.
